# Application of power battery under thermal conductive silica gel plate in new energy vehicles

**DOI:** 10.1038/s41598-023-43388-0

**Published:** 2024-01-03

**Authors:** Hang Ma, Shirong Zong, Banglong Wan, Guodong Wang, Qiang Tian

**Affiliations:** Yunnan Yuntianhua Co., Ltd., Kunming, 650228 China

**Keywords:** Engineering, Materials science, Mathematics and computing, Nanoscience and technology, Optics and photonics, Physics

## Abstract

This study aims to improve the performance of automotive battery thermal management systems (BTMS) to achieve more efficient heat dissipation and thus reduce hazards during driving. Firstly, the research parameters and properties of composite thermally conductive silicone materials are introduced. Secondly, the heating principle of the power battery, the structure and working principle of the new energy vehicle battery, and the related thermal management scheme are discussed. Finally, the research results are presented from the experimental test and controller design. In addition, to achieve the research goal, the composite thermally conductive silica gel plate (CSGP) material is studied in detail and parametrically analyzed, and the heating mechanism of the power battery is discussed in depth. The temperature characteristics after adding CSGP are experimentally tested, and the controller of the BTMS of the new energy vehicle is designed, including hardware circuits and software modules. The findings show that the temperature characteristics of the battery module have obvious limitations without CSGP. When the battery module operates at a 4C magnification, the temperature exceeds the safety threshold by 38.4%, with particular potential safety risks. Then, the maximum temperature of the battery module with CSGP can be controlled within 50 °C, and the temperature characteristics are prominently improved. Lastly, the controller of the BTMS is tested, and the results reveal that it has remarkable voltage recovery ability. According to the research results, the performance of automotive BTMS can be significantly improved, and better heat dissipation can be effectively achieved by adding CSGP. This helps reduce the hazards of driving. Moreover, the designed controller performs well in voltage recovery, providing solid theoretical support for further developing the CSGP battery management system.

## Introduction

To better explore the thermal management system of thermally conductive silica gel plate (CSGP) batteries, this study first summarizes the development status of thermal management systems of new energy vehicle power batteries to lay a foundation for subsequent research. A good ecological environment might drive the fairest social product and the most inclusive of people’s well-being. In the contemporary context, the previous developmental paradigm that prioritized economic gains at the expense of the environment no longer aligns with the imperatives of today's emphasis on preserving pristine natural resources and ensuring sustainable progress. To create temperate waters and lush mountains in the new era is the need to build a beautiful China and the primary goal of meeting people’s needs for a good life^1^. With the intensification of air pollution and the depletion of fuel resources, developing efficient, environmentally friendly, and green energy drives in transportation has become an inevitable trend and change^2^. In recent years, the pursuit and proliferation of electric and hybrid electric vehicles have emerged as efficacious measures to address these challenges. However, there are still some bottlenecks. Foremost among these is the thermal safety performance of power batteries, which constitutes the primary hindrance to the widespread adoption of electric and hybrid electric vehicles^3^.

The power battery module is the core component of the energy supply, and its safety assessment, management, and protection have received extensive attention in recent years^4^. Among various rechargeable batteries such as lead-acid, sodium-sulfur, and lithium-ion batteries, lithium-ion batteries have many merits, such as elevated energy density, substantial specific capacity, minimal environmental impact, stable functionality, and prolonged cycle life^5^. Therefore, it is also considered the most suitable power source for new energy vehicles^6^. Nevertheless, the operating temperature of lithium-ion batteries will significantly affect physical and chemical properties, such as cycling stability, safety performance, and service life^7,8^. Particularly noteworthy is the occurrence of thermal runaway during high discharge rates, a phenomenon triggered when temperatures exceed the typical operational threshold of lithium-ion batteries, significantly impinging on their performance^9,10^. Hence, it is necessary to explore an effective thermal management system for power battery modules to develop and popularize new energy vehicles well and improve the safety of new energy vehicles. In addition, the battery operating temperature will be maintained within a reasonable range.

In recent years, with the rapid development of new energy vehicle technology, the performance of the battery thermal management system (BTMS) is crucial to ensure battery safety, life, and performance. In this context, researchers continue to explore new heat dissipation methods to improve the heat dissipation efficiency of battery modules. Compared with traditional heat dissipation methods, CSGP, as a new thermal conductivity material, is gradually attracting more and more attention. CSGP has many advantages, making it a broad application prospect in battery thermal management (BTM). First, compared with traditional heat dissipation methods, CSGP has excellent thermal conductivity, which can quickly transfer the heat generated by the battery from the battery body to the heat dissipation area, effectively reducing the battery temperature. Its thermal conductivity is expected to provide better temperature control at high-rate discharges, thereby improving the stability of the battery system under extreme conditions. Second, applying CSGP also helps improve the battery module's temperature uniformity. Due to its thermal conductivity, CSGP can achieve a more uniform heat distribution and reduce the generation of hot spots, thereby reducing the risk of local overheating of the battery module and extending the battery’s service life. Regarding BTMS performance, introducing CSGP is conducive to improving several vital aspects. Firstly, by effectively controlling the battery temperature, the temperature rise caused by heat accumulation during high-rate discharge can be reduced, thus enhancing the safety of the battery system. Secondly, CSGP is expected to improve battery modules' heat conduction efficiency, helping maintain stable battery performance under long-term high-load use.

The literature shows that the current research on thermally conductive silicone materials and new energy vehicle BTMS has achieved specific development. But there needs to be more information on combining the two to study the thermal management performance of vehicle batteries. Based on this, this study first gives the composite thermal conductive silicone, the principle of battery heat generation, and the structure and working principle of the new energy vehicle battery. Then, the battery heat generation theory and the new energy vehicle battery are combined to give the BTM scheme of a new energy vehicle. Lastly, automobile batteries' thermal management performance and temperature characteristics are tested and analyzed in design test experiments. The innovation is that the BTMS controller is designed based on the structure and working principle of the automotive battery. Besides, some of its performance is tested. This study aims to improve the thermal management performance of new energy vehicle batteries and provide ideas for the continuous development of BTM technology. The core goal of the study is to optimize thermal management systems for automotive batteries to achieve more effective heat dissipation strategies, thereby reducing potential risks that may arise during driving. The thermal dissipation mechanism of power batteries is analyzed in depth by studying the performance parameters of composite thermally conductive silicone materials, and BTM solutions and controllers for new energy vehicles are innovatively designed. It is found that introducing new technologies such as CSGP can significantly enhance the performance of BTMS, effectively improve the heat dissipation efficiency, and thus reduce the potential risks during driving.

## Literature review

Domestic and international researchers have devised diverse cooling methodologies utilizing BTMS to address thermal runaway incidents in power batteries. According to the different heat transfer media, it can be divided into air-cooled, liquid-cooled, and phase change material management systems. Furthermore, there are active and passive cooling management systems based on the form. Passive cooling systems encompass phase change materials, heat pipes, and natural convection management systems. Active cooling systems incorporate liquid cooling, forced convection, and hybrid secondary cooling systems. For instance, Jithin and Rajesh^11^ proposed a novel reverse-layered airflow battery heat dissipation model^11^. The flow channel was classified into multiple layers of fluid flow channels. The cooling fluid flowed in the opposite direction in adjacent flow channels. The heat was transferred through transverse separators for heat conduction and convection. This enhanced the heat dissipation capability of the battery module. For the problem of uneven air inlet and outlet temperature in cylindrical lithium-ion battery modules, Wu et al.^12^ innovatively devised a novel BTMS, leveraging an air distribution tube as its foundation^12^. Bernagozzi et al.^13^ proposed a novel air-cooling strategy by incorporating spoilers within the airflow distribution chamber of a parallel air-cooling model, thereby enhancing the battery cooling system's heat dissipation efficacy^13^.

The above review showed that domestic and international researchers had adopted various thermal management strategies for thermal runaway events in power batteries, including liquid-cooled, air-cooled, and phase change material management systems, and active and passive cooling management systems. Although these studies progressed in thermal management, some things still needed improvement. Firstly, some methods may have significant effects under specific conditions but perform poorly in other situations. Secondly, some studies may lack comprehensive consideration of the performance of battery modules under various operating conditions, leading to potential limitations in practical applications. In addition, existing methods may face challenges in terms of energy consumption and other aspects. In contrast, this study aimed to significantly improve the performance of automotive BTMS by introducing new technologies such as CSGP to achieve more effective heat dissipation, thereby reducing driving risks. Unlike the previous literature, this study comprehensively considered the application of CSGP and the design of system controllers to solve thermal runaway problems. Through this comprehensive approach, this study can fill the gap in existing studies that may have unstable effects under specific conditions, as well as in the overall consideration of complex working conditions.

## Theoretical basis and method construction research

### Thermal conductive silica gel and power batteries for new energy vehicles

**Figure 1:**
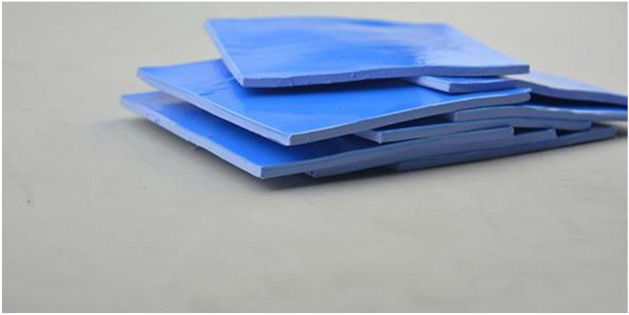
Thermally conductive silica gel plate.

As a high-end thermal conductive composite material, the thermal conductive silica gel has been widely used in new energy vehicles. The thermal conductive adhesive sealant is considered a single component with good thermal conductivity, room temperature curing silicone sealant^14^, and excellent thermal conductivity. The finished sheet of thermal conductive silica gel is presented in Fig. 1. By condensation reaction of moisture in the air, thermal conductive silica gel can achieve low molecular release, initiate crosslinking and curing^15^, and form high-performance elastomers. The thermal conductive silica gel not only has high and low-temperature resistance, but also has the advantages of aging resistance, electrical insulation, etc. In addition, it also exhibits excellent moisture resistance, impact resistance, corona resistance, leakage resistance, chemical stability, and good adhesion with most metal and non-metallic materials^16^. The thermal conductive silica gel has broad application prospects in various fields, and the relevant parameters are detailed in Table 1^17^. At the same time, thermal conductive silica gel plays a vital role in improving the range and safety of new energy vehicles. Currently, the battery systems used in new energy vehicles mainly include different types such as lithium iron phosphate, lithium manganese oxide, ternary batteries, and fuel cells, and the number of battery cells directly affects the vehicle's endurance. As the number of cells increases, the distance between cells is smaller. Nevertheless, battery cells generate much heat during discharge or charging. If they cannot effectively dissipate heat, it can easily lead to accidents such as battery cell short circuits and fires. In this case, thermal conductive silica gel, as a flexible and ductile material, can evenly fill the cell gaps and quickly transfer heat to the cooling area or outside air. Thus, the safety of the battery system is ensured, maximizing the advantages of the number of batteries and effectively improving the endurance of the power battery system of the new energy vehicle. Besides, in different cooling methods, thermal conductive silica gel acts as a bridge of heat transfer, playing a key role between the cells and the heat dissipation area, achieving efficient heat transfer. Its insulation performance can effectively prevent strong voltage caused by excessive current in the battery cell, ensure the normal operation of the power battery system, and avoid faults such as short circuits.

**Table 1:** Parameters of thermally conductive silica gel.

Parameter name	Unit	Specific description or indicator value
Shape		White paste
Curing category		Dealcoholized
Elongation	%	200
Shear strength	MPa	Greater than or equal to 2.5
Peel strength	N/mm	Greater than 5
Proper temperature	°C	60–280
Linear shrinkage	%	0.3
Dielectric constant	MHz	1.2
Dielectric strength	KV/mm	21

### Theory of battery heat production

The previous section analyzes the theory of thermally conductive silicone. The results indicate thermal conductive silicone has good thermal conductivity and chemical characteristics. It is often used as a thermally conductive material for BTMS. The principle of heat generation of automotive batteries will be introduced in this section to explore the thermal management system of automotive batteries.

**Figure 2:**
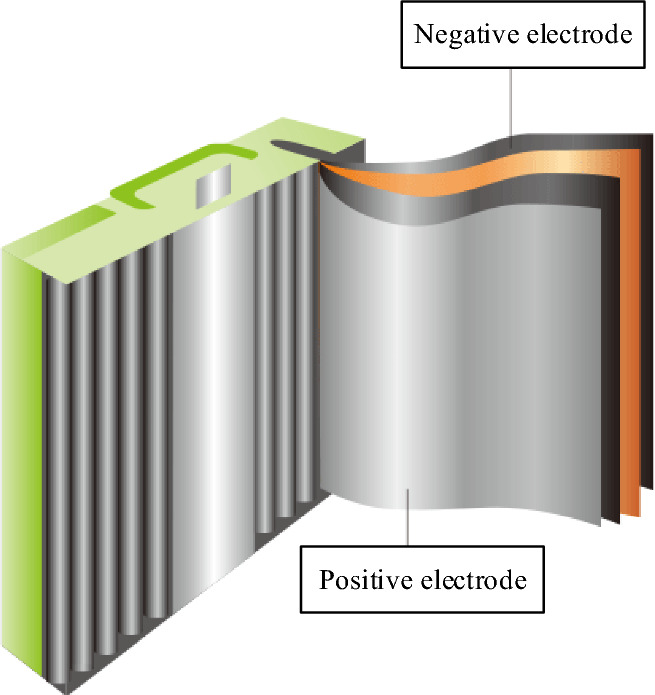
Structure of common lithium-ion batteries.

During the operation of lithium-ion batteries, chemical reactions occur between the internal materials, generating a large amount of heat. This will cause the overall temperature of the battery to rise sharply, resulting in the attenuation of the battery capacity. The battery’s overall performance declines and even thermal runaway problems occur. The heat generation principle of lithium-ion batteries is analyzed to provide a theoretical basis for the subsequent heat generation simulation of battery modules and the design of BTMS^18^. The structure of lithium-ion batteries commonly used in the market is plotted in Fig. 2.

The heat-generating reactions of lithium-ion batteries primarily encompass processes such as the decomposition of the solid electrolyte interface film, electrolyte decomposition, positive electrode decomposition, reactions involving the negative electrode and electrolyte, interactions between the negative electrode and binder, and the Ohmic heat attributed to internal resistance within the battery^19^. During the charging and discharging process of the lithium-ion battery, lithium ions move back and forth between the positive and negative electrodes, accompanied by the central internal reaction heat, side reaction heat, polarization heat, and Ohmic heat. The cumulative heat production emerges from the summation of these aforementioned components^20^.

The heat generation of the battery is mainly affected by the nature material of the battery itself. Different cathode materials, charge–discharge rate, charge–discharge cycle mode, external environment, and other internal mechanisms of the battery will affect the heat production of the entire battery^21^. Therefore, the control variable method must be adopted when the heat production of the battery is tested. The external conditions, such as the external ambient temperature, are tightly regulated to ensure consistency in battery capacity when applying a specific battery type. Then, the charging and discharging process at different rates is carried out. For example, a fully charged battery cell is positioned within a thermally insulated environment, and its discharge ensues at a rate of 2C until the cut-off voltage is attained, spanning 1800s. The heat production rate of the battery cell is calculated by measuring the heat produced during the entire discharge process^22^.

In the process of using the lithium iron phosphate power battery, the heat generation is considerably huge due to the charging and discharging. The battery temperature will rise if the heat is not dissipated in time^23^. The heat emanating from the battery comprises several components, including ${Q}_{rea}$, originating from internal chemical reactions, the polarization heat ${Q}_{act}$, attributed to polarization resistance-induced polarization heat, ${Q}_{ohm}$, resulting from current flow-induced Joule heat, and ${Q}_{sid}$, stemming from side reactions during overcharge and over-discharge. Here, *Q* represents the total heat production of the battery. The final heat production equation for a lithium-ion power battery reads:

1 $$Q={Q}_{rea}+{Q}_{act+}{Q}_{ohm}+{Q}_{sid}$$

The average heat production rate of the power battery can be calculated according to Eq. (2).

2 $$q=\frac{Q}{t}$$

*q* represents the average heat production rate of the battery, in *W*. *Q* refers to the total heat production generated by the lithium-ion power battery during the working process, and the unit is *J*. *t* stands for the active time of the lithium-ion power battery, in *s*.

The specific heat capacity of the power battery is a vital parameter essential for calculating battery heat production^24^. Two strategies are applicable for measuring specific heat capacity. One is experimental measurement, while the other involves calculating the average specific heat capacity by disassembling the battery and analyzing its chemical composition^25^. The experimental equation strategy can generally measure the specific heat capacity. First, the battery is placed in an incubator to set the initial temperature. The battery is discharged at a 4C discharge rate to obtain the heat *Q* generated from the beginning to the end of the discharge. The battery temperature (T1 and T2) is recorded at the beginning and end of the work. Then, the specific heat capacity of the battery is obtained according to Eq. (3).

3 $$Q={C}_{p}m({T}_{2}-{T}_{1})$$

It can also be expressed as:

4 $$q={C}_{p}m\frac{dT}{dt}$$

In Eq. (4), *m* represents the mass of the battery.

**Figure 3:**
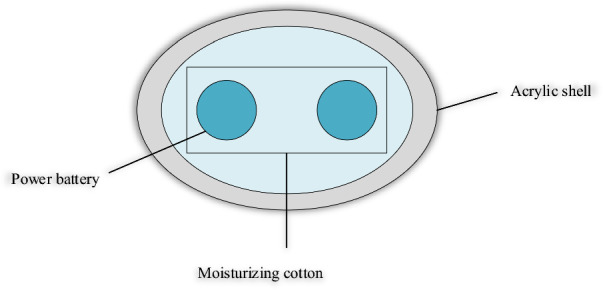
Cross-sectional view of battery heat generation test system.

The experimental power battery heat generation method uses a square iron-shell lithium iron phosphate power battery^26^ with a capacity of 20Ah. The testing procedure can be described as follows. Two thermocouples are distributed on the central surface of the square iron-shell lithium iron phosphate power battery. The other end of the galvanic couple is connected to Agilent. The recording time step is set at 2 s. Meanwhile, the battery is tightly wrapped in black thermal insulation cotton (rubber-plastic foam closed-cell sponge). The fully charged battery is placed in a box constructed of acrylic panels to create a nearly insulating environment. Then, the battery is discharged at a 4C rate. The battery temperature data detected by Agilent is exported through the computer for analysis and calculation after discharge. A cross-sectional view of the battery heat generation test system is denoted in Fig. 3.

Figure 3 demonstrates that in the battery heat generation test system, the outermost layer is composed of an acrylic shell, followed by insulation cotton with a thickness of 10 mm. The innermost is composed of a power battery.

During the heat generation experiment, the battery will exchange three forms of heat with the external environment: heat conduction, heat convection, and heat radiation. Therefore, the heat generated by the power battery during the discharge process will increase the battery temperature and release heat to the ambient environment^27^. According to Boltzmann’s law of thermal radiation, the temperature difference between the battery and the external environment is minimal. Hence, the thermal radiation effect can be neglected. The convective heat transfer coefficient in a natural convection environment is 5–25 W m^−2^ K^−1^. Thus, the heat generation equation of the battery discharge process is as follows.

5 $$Q={Q}_{1}+{Q}_{2}+{Q}_{3}$$

In Eq. (5), the power battery’s total heat when discharged at 4C is *Q*. The heat used by the battery itself is ${Q}_{1}$. The heat dissipated by the convective heat exchange between the battery and the external environment is ${Q}_{2}$. The battery’s heat for radiative heat exchange with the external environment is ${Q}_{3}$.

A simulation study is performed on the temperature characteristics of the used LiFePO4 battery at 4C^28^. Employing the data equation fitting function within Origin software, a fitting curve of battery temperature is derived from the temperature data collected during the power battery's 4C discharge rate testing. Besides, the corresponding relationship is established. The relationship between temperature and time of the fitted curve is:

6 $$T=at+b$$

In Eq. (6), *T* represents the average temperature of the square lithium iron phosphate power battery. *t* means the discharge time of the square lithium iron phosphate power battery. According to Eqs. (4)–(5), assuming that the radiation heat transfer between the power battery and the external environment is zero, the convection heat transfer coefficient with the external environment is 5*W m*^−2^·*K*^−1^, *a* = 0.01593, *b* = 28.41552, the average heat production rate of the square iron-shell lithium iron phosphate power battery at the 4C discharge rate is 27.81 W.

### New energy vehicle battery working principle and thermal management scheme

The previous section analyzes automobile batteries' heat generation principle, involving the batteries' heat generation calculation and related experimental research. Then, in this section, the thermal management scheme of automotive batteries will be built based on the principle of battery heat generation and combined with the working principle of new energy vehicle batteries.

New energy vehicles rely on batteries as their primary power sources. Lead-acid and nickel-metal hydride batteries consider factors such as battery cost, power ratio, cycle life, and manufacturing process compared with lithium-ion batteries^29^. Lithium batteries have become the main choice for the next generation of new energy vehicles due to their high energy density and battery life. However, the continued advancement of lithium-ion batteries for new energy vehicle battery packs may encounter substantial constraints posed by temperature and safety considerations.

**Figure 4:**
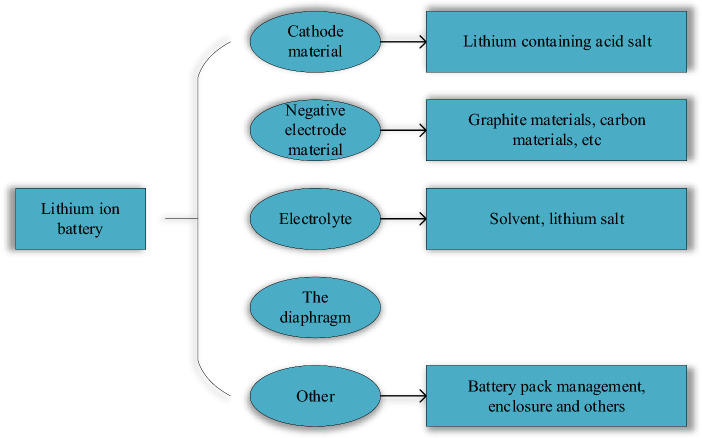
Schematic diagram of the basic structure of lithium battery.

Although lithium batteries are designed into different shapes and structures according to different needs, they are all composed of positive and negative electrode sheets, electrolytes, separators, and shells^30,31^. The schematic diagram of the basic structure is illustrated in Fig. 4.

The separator is located between the positive and negative electrode sheets to separate the positive and negative electrodes of the battery to avoid a short circuit. It allows the free passage of lithium ions (Li^+^) but prevents the free path of electrons (e^−^). Acting as the conduit for positive and negative ions, the electrolyte plays a pivotal role, typically in liquid or solid form. The electrolyte must exhibit stable chemical properties and high electrical conductivity, influencing factors like battery temperature, performance, and safety considerations.

**Figure 5:**
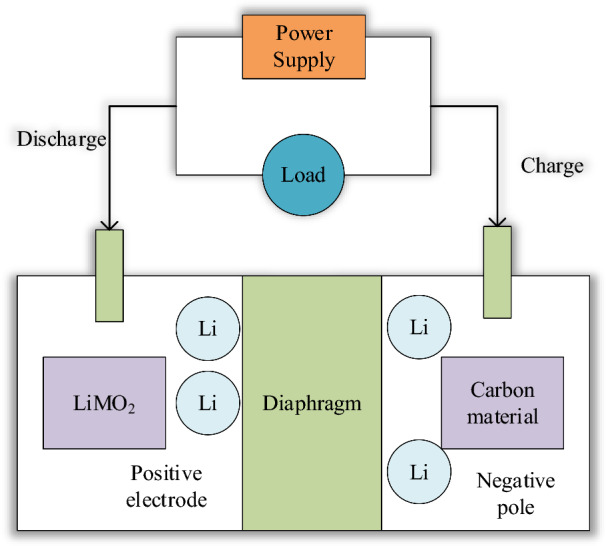
Thermal working principle of lithium battery.

Lithium batteries' positive electrode contains an active material composed of lithium-containing metal oxide, while the negative electrode consists of intercalated lithium carbon (Li_x_C). Lithium batteries’ charging and discharging process is essentially the intercalation and extraction of lithium ions in positive and negative active materials. Figure 5 reveals the working principle.

**Figure 6:**
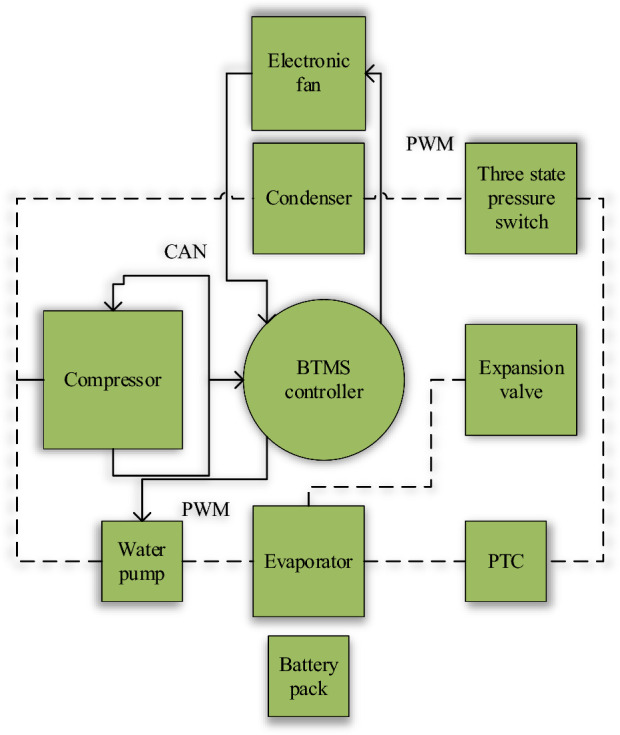
Overall structure of thermal management system.

The BTMS is mainly divided into two cycles^32^. One way is the preheat cycle. The temperature sensor is placed at the water inlet to detect the water temperature of the water inlet of the electronic water pump. Another temperature sensor is situated at the water outlet to gauge the water temperature at the outlet of the electronic pump. The Positive Temperature Coefficient (PTC) heater receives Controller Area Network (CAN) information through the external interface power supply. When the battery pack temperature is too low, the heat generated by the PTC heats the water flowing through it. The heated water is then circulated through the battery pack, elevating its temperature. The PTC heater stops working when the battery pack temperature is too low. Under the pump's influence, cool water circulates through the battery pack, reducing its temperature. The other way is the refrigerant cycle. This is similar to the cooling principle of an air conditioner. The refrigerant changes between gaseous, liquid, high-pressure, or low-pressure^33^. From the outside, the refrigerant passes through the compressor, condenser, expansion valve, and evaporator, constantly releasing or absorbing heat. Its complete thermal management system structure is suggested in Fig. 6.

Figure 6 presents that the structure of the automotive BTMS is principally composed of a BTMS controller, compressor, condenser, evaporator, and other modules. These modules are connected by inlet and outlet pipes, controlled by expansion valves, pumps, and three-state pressure switches.

### Thermal management performance test of vehicle battery based on composite thermally conductive silica gel plate (CSGP) coupled with air cooling

The previous section proposes the working principle of new energy vehicle batteries and BTM schemes. But the battery’s temperature can rise during the charging and discharging process or exposure to the sun. When the battery temperature is above the appropriate operating temperature for a long time, battery life and safety will be reduced. If the battery’s temperature is not reasonably controlled within the proper temperature range, it will also lead to thermal runaway and cause unnecessary safety hazards. Given the large amount of heat generated by the battery during the charging and discharging process, the excellent thermal conductivity and heat dissipation performance of CSGP are employed to take away the heat in the module in time by combining air cooling. Thus, in this section, experiments will be designed to test the thermal management performance of automotive batteries based on CSGP-coupled air cooling.

In addition, during the experiment, it is also necessary to pay attention to the influence of thermal resistance between the battery body and CSGP. Thermal resistance is an important factor in the heat conduction process, affecting the battery module's temperature distribution and heat dissipation effect. Although CSGP has excellent thermal conductivity, there is still a certain thermal resistance between the battery body and CSGP, which may affect the experimental results. However, in this study, the main focus is to explore the performance of CSGP in terms of heat dissipation in battery modules. Although there may be a certain thermal resistance between the battery body and CSGP in practical applications, this experiment has not thoroughly studied this factor. The goal is to preliminarily evaluate the potential of CSGP in heat dissipation and provide better temperature control for battery modules under high-rate discharge conditions.

**Figure 7:**
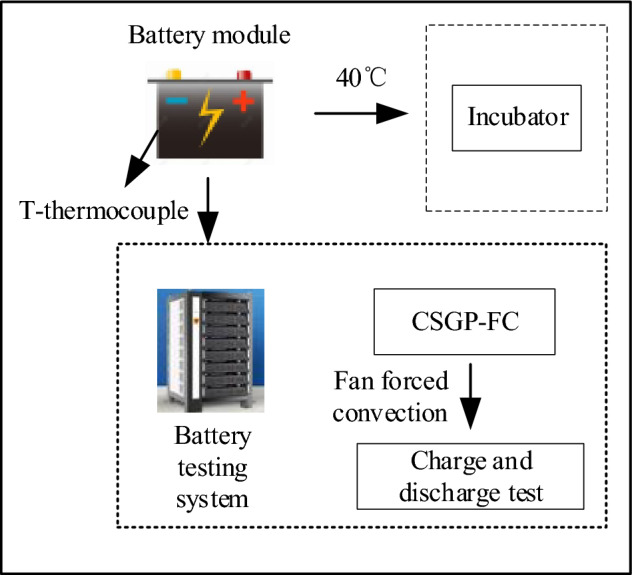
Schematic diagram of CSGP-FC experimental test platform.

The platform assembly for the experimental test is displayed in Fig. 7. Battery modules with different cooling systems are separated into the incubator. The operating temperature is precisely set at 40 °C, and the battery module will be at this temperature for experiments. The experimental temperature is set at 40 °C. The common power battery testing environment requirements are between 0 and 40 °C. If the ambient temperature is lower than 0 °C or greater than 40 °C, the performance of the power battery will decrease, resulting in a corresponding decrease in discharge capacity. Battery modules will be placed in an incubator for 1 to 2 h to ensure the accuracy of the experiment. Following temperature stabilization, the battery modules undergo charging and discharging cycles via a battery test system. One end of the T-type thermocouple is attached to the surface of the battery, and the other is connected to the Agilent temperature inspection instrument to record the temperature change value of the battery module. The Agilent operates at a two-second interval, meaning the battery temperature is recorded every two seconds. In addition, composite thermally conductive silica gel plate-forced cooling (CSGP-FC) modules are forced convection by fans. The direct-current power supply provides the fan’s energy. It is necessary to charge and discharge each battery before the experiment and test its internal resistance and charge–discharge voltage curve to ensure the accuracy of the experiment. The battery module is assembled utilizing cells with closely matched internal resistances, with specific attention paid to equalizing the state of charge across all batteries.

The charge and discharge process parameters are set, as outlined in Table 2. It shows the test parameters during charging and discharging. The battery module is charged with the constant current at 20A at a cut-off voltage of 18.25 V before the experiment starts. The battery module assesses this continuous voltage until the current drops to 0.2A. The battery module will be discharged at the discharge rate of 1C, 2C, 3C, and 4C, respectively, after it stands for 30 min. The battery module will rest for another 30 min when the battery’s voltage drops to 10 V.

**Table 2:** Charge/discharge steps.

Run process	Constant current charging	Constant voltage charging	Rest 30	Discharge process	Cyclic process
Time/time			30 min		10
Current size /A	20	The cut-off current is 0.2A		20/40/60/80	
Voltage /V	Cut-off voltage 18.25	18.25		Cut-off voltage 10	

The heat dissipation and temperature equalization ability of the CSGP to the battery module is tested. The battery module design is as follows. First, the battery and the thermocouple are connected. The thermocouples of the central battery are arranged at the center of the battery and near the battery, and the front and back sides are arranged as such. The remaining four cells are arranged only in the center of the battery, with 12 thermocouples in one module. The CSGP sizes used here are 100 × 140 × 5 mm (without fins) and 141 × 140 × 5 mm (with fins). The fin-equipped CSGP maintains an equivalent height to that of the battery, while the overall width is extended by 21 mm, with protruding fins adding an extra 10.5 mm on either side. This design increases the convective heat transfer area, facilitating enhanced heat dissipation. Additionally, the protruding sides are thickened by 5 mm, and the inner width is just enough to clamp the battery. The contacting surface of the CSGP and battery is maximized to optimize heat transfer efficiency. The battery module comprises five square lithium iron phosphate batteries connected in series. Each battery has two CSGPs for heat dissipation, for a total of six CSGPs. Subsequently, the cooling effect between different thermal management methods is compared. Three modules without fins are designed for charge and discharge experiments: CSGP-FC module (c), CSGP-FC module (d), and pure battery module (e) by changing the fins of CSGP, forced convection, and the wind speed of forced convection. In addition, thermal management system heat dissipation is studied.

### Design of BTMS controller based on CSGP coupled with air cooling

The previous section tested and analyzed the thermal management performance of automotive batteries based on CSGP-coupled air cooling. Therefore, in this section, the BTMS controller will be designed, including software and hardware design, to enhance the thermal management effect of automotive batteries based on CSGP coupled with air cooling.

**Figure 8:**
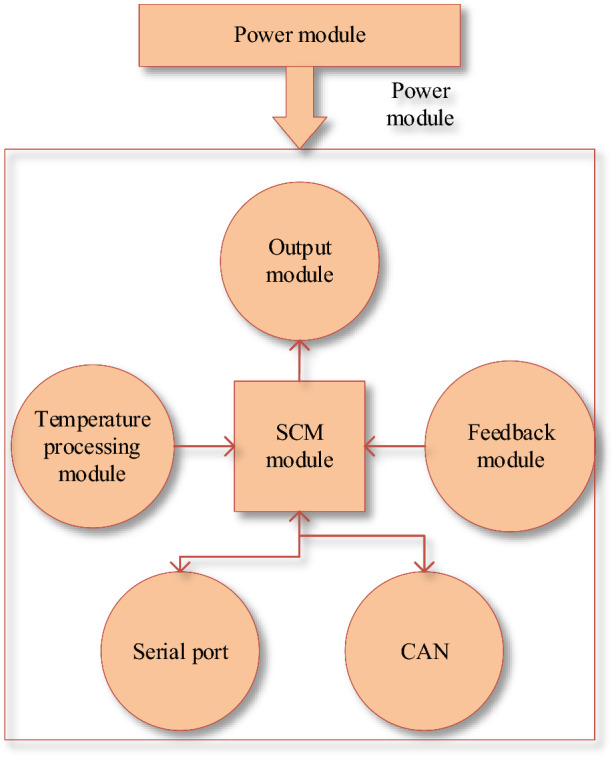
Controller hardware circuit structure diagram.

The hardware circuit of the BTMS controller mainly includes a power supply module, a temperature processing module, a drive module, a feedback module, a microcontroller module, and a CAN communication module. The hardware circuit structure diagram is shown in Fig. 8.

Figure 8 depicts that the hardware circuit of the BTM controller is mainly composed of a single-chip microcomputer module, a temperature processing module, a feedback module, an output module, and a power module. The single-chip microcomputer module is connected to the serial port and CAN communication.

The power supply voltage of 24 V DC is converted to 5 V DC to power other modules.

The voltage value across the temperature sensor is collected. The voltage value is sent to the microcontroller’s Analog to Digital Converter (ADC) port for sampling after it passes through the amplifier.

The drive module controls the speed of the electronic fan and the electronic water pump.

The feedback module receives the fault feedback signal of from the electronic water pump and the electronic fan. It also gets the three-state pressure switch's high, medium, and low-pressure states. After processing these states, the signal is transmitted to the microcontroller.

The single-chip microcomputer module performs ADC sampling on the voltage value collected by the temperature sensor. The standard 1/0 port reads the fault feedback signal of the electronic fan and electronic water pump. It outputs the pulse width modulation control signal. It can also carry out CAN communication and serial communication.

CAN communication controls compressor and PTC. It bridges the single-chip microcomputer and the external information exchange.

**Figure 9:**
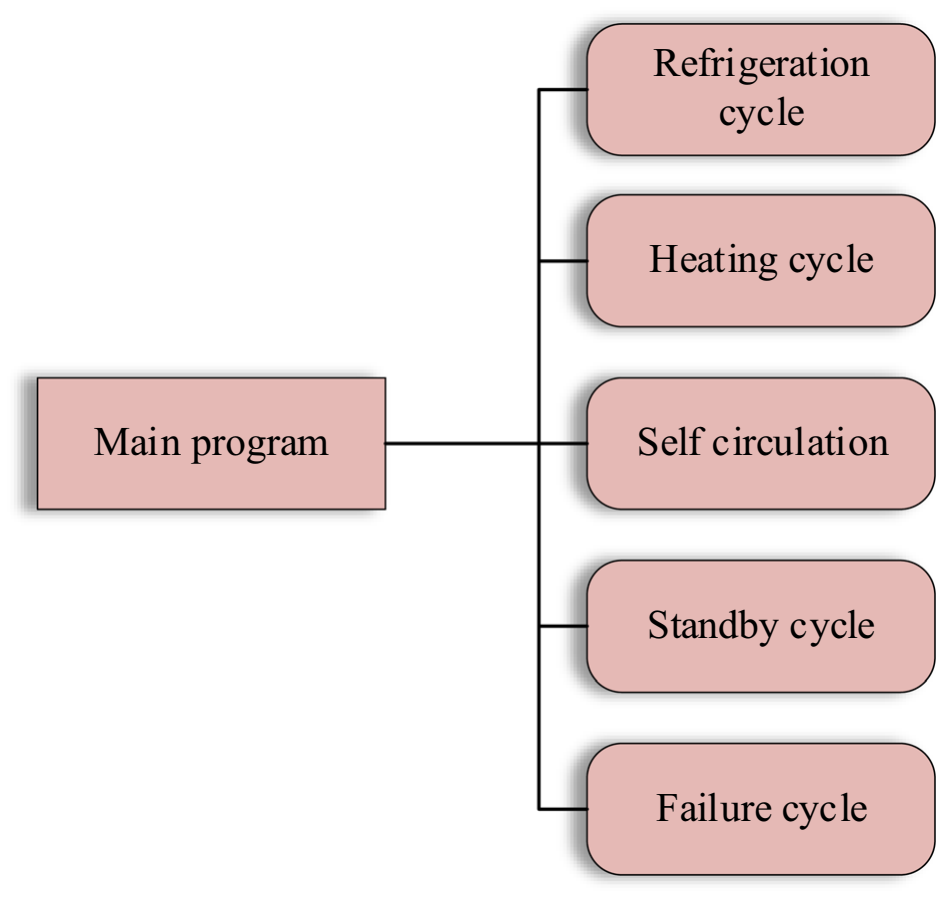
Software structure diagram.

The BTMS controller software structure encompasses multiple functions. It is entered by the main program and powered up by the system. The BTMS controller primarily determines the cycle mode based on the message information received from the battery pack. If the message information is not received, the failure loop is entered. After receiving the message information, the controller will enter the refrigeration, heating, self-circulation, and standby cycle according to the battery pack’s temperature. In each cycle mode, the current battery pack temperature is continuously judged. If the temperature deviates from the designated range, the sub-cycle is exited, and the main program is re-entered for temperature assessment and selection of the next sub-cycle. The software structure is drawn in Fig. 9.

The main program continuously judges the temperature and selects to enter the corresponding sub-cycle.

The system needs compressor cooling to reduce the temperature of the battery pack when the temperature of the battery pack is too high. The compressor's speed is modulated based on the temperature gradient to achieve varying degrees of cooling.

The system needs a PTC heater for heating to increase the temperature of the battery pack when the temperature of the battery pack is too low. The PTC heating power is controlled according to the temperature gradient to achieve different heating effects.

The temperature of the battery pack is within a relatively appropriate range. At this time, turning on the water pump and using the water circulation to take away part of the waste heat is essential.

Upon system initialization, all components remain inactive. If the message is invalid, the BTMS controller cannot receive the temperature information from the battery management system and cannot make a judgment based on the actual temperature of the current battery pack. However, the unit can operate normally, and the cycle mode can also be selected according to the temperature of the water outlet. Once the message is re-established, the failure mode is exited.

Furthermore, when the BTMS controller fails and enters the fault loop, the BTMS controller will take a series of measures to repair the fault to safeguard the normal operation of the BTMS. First, the controller checks the power module to ensure that it provides a stable supply voltage conversion. They will then check the temperature handling module to ensure the temperature sensor works accurately and properly. If the drive module fails, the controller will investigate the speed control of the electronic fan and the electronic water pump and may need to repair or replace the drive module. The feedback module is responsible for receiving the fault feedback signal, so the controller will check whether this module is working properly and whether the fault information is accurate. The microcontroller module is the brain of the entire system. The controller checks that the connection of the microcontroller module is stable to ensure that it can perform ADC sampling, control output, and communication functions. Ultimately, the controller checks the CAN communication module to guarantee that it is transmitting information properly. During the repair process, if any module is found to be faulty, the controller may take appropriate repair, replacement, or adjustment measures to restore the normal operating condition of the BTMS controller. This comprehensive approach to maintenance and repair will help ensure the stability and reliability of the BTMS, thereby safeguarding the performance and safety of the entire battery system.

## Research result

### Analysis of battery thermal management performance of CSGP coupled with the air-cooled system

(1) Temperature characteristics of battery modules under natural convection conditions

**Figure 10:**
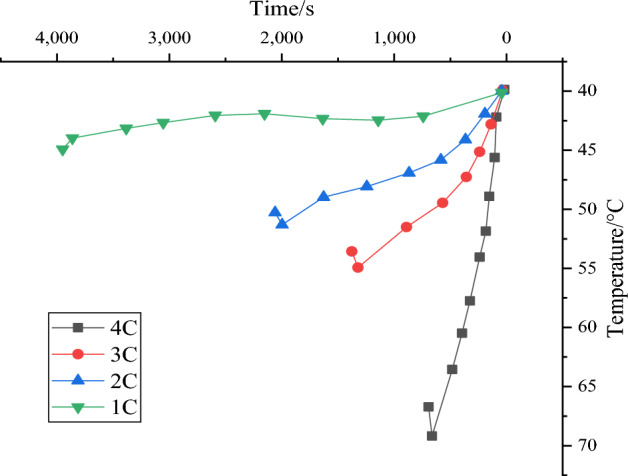
Temperature graph of battery temperature as a function of time during different discharge processes.

The temperature characteristics of the battery module by the CSGP under natural convection conditions are obtained using the experimental verification platform designed in “Section Thermal management performance test of vehicle battery based on composite thermally conductive silica gel plate (CSGP) coupled with air cooling”, as portrayed in Fig. 10.

Figure 10 details that the temperature of the battery module without CSGP has a very different heat generation with an increased discharge rate. The larger the discharge rate might result in more heat accumulated in the central battery, the steeper the temperature curve, and the greater the battery temperature rise. The maximum battery temperature reaches 45 °C, 51.5 °C, 55 °C, and 69.2 °C when the battery module is discharged at rates of 1C, 2C, 3C, and 4C, respectively. When the maximum temperature of the battery is at a 1C discharge rate, the allowable operating temperature of the battery maintains below the safety temperature of 50 °C, thereby the thermal management strategy is unessential. However, discharges at 2C, 3C, and 4C rates exceed the recommended temperature range for safe battery operation. Especially under the condition of 4C, the battery temperature exceeds the safe temperature value by 38.4%, far exceeding the safe temperature allowed by the battery. This will seriously affect the cycle life and safety of the battery. If the battery works at an irregular temperature with a long-time operation, it will cause a safety hazard in severe cases. These findings highlight significant limitations in the temperature characteristics of standard battery modules.

(2) Research on thermal control characteristics of CSGP coupled natural convection system in battery modules.

**Figure 11:**
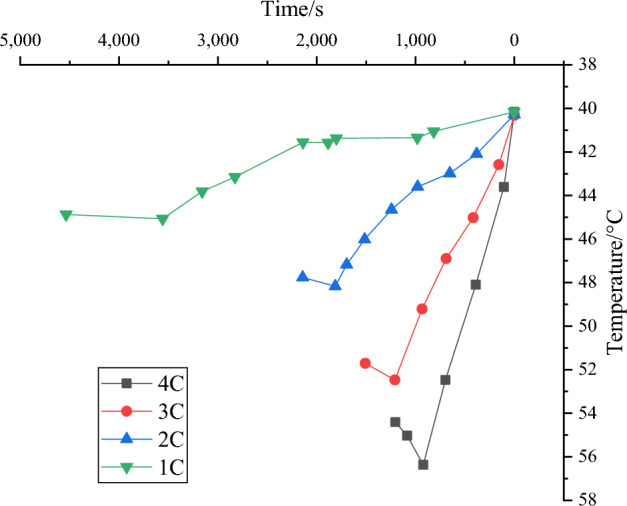
Thermal control characteristic results of CSGP coupled natural convection system in the battery module.

The thermal control characteristics of the battery module's CSGP coupled natural convection system are obtained using the experimental verification platform designed in “Section Thermal management performance test of vehicle battery based on composite thermally conductive silica gel plate (CSGP) coupled with air cooling”, as demonstrated in Fig. 11.

The battery module with the CSGP can reach 44.8 °C, 47.9 °C, 52.5 °C, and 56.3 °C, respectively, after discharge at 1C, 2C, 3C, and 4C rates under natural convection conditions. The addition of CSGP greatly helps battery heat dissipation compared with Fig. 10 without any cooling measures. Without forced convection, the maximum temperature for the 2C discharge rate remains below 50 °C. The cooling effect of 3C and 4C is good. The temperature curve has slowed down compared to no cooling measures. The maximum temperature decreases by 2.5 °C and 12.9 °C, respectively. The heat dissipation efficiency is improved by about 4.74% and 22.9%. This indicates that CSGP is suitable for solving the severe high-temperature problem of batteries due to its high thermal conductivity.

Additionally, in the above experiments, it is found that the temperature of the battery module with CSGP in the case of high-rate discharge exceeds the optimal operating temperature range of lithium-ion batteries. This is because high-rate discharge causes more heat to be generated inside the battery, and CSGP as a heat conductor will help transfer this heat, but may cause the temperature to rise. Meanwhile, natural convection conditions may be relatively weak and may not be able to dissipate heat from the battery module quickly and efficiently, resulting in an increase in temperature. Despite the above situation, it can still be observed from the experimental results that the introduction of CSGP has played a significant role in improving the heat dissipation of the battery. Compared with the case without any cooling measures, the addition of CSGP greatly improves the heat dissipation effect of the battery module. Especially in the absence of forced convection, the maximum temperature of 2C discharge rate can be controlled within 50 °C, and the cooling effect is also good at 3C and 4C rates. Compared to the temperature curve observed without cooling measures, the maximum temperature decreased by 2.5 °C and 12.9 °C, respectively. At the same time, the heat dissipation efficiency has also been Notably improved, about 4.74% and 22.9%, respectively. These results reveal that despite the temperature exceeding the optimal operating range, CSGP has potential applications in solving high temperature problems in batteries due to its excellent thermal conductivity.

### BTMS controller performance

**Figure 12:**
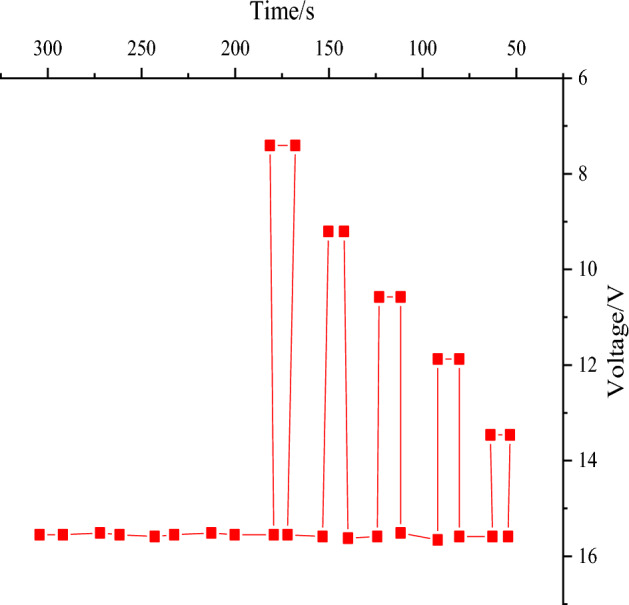
Reset function test results for voltage dips.

The voltage dip reset function test is performed on the BTMS controller, and the test results are revealed in Fig. 12.

Figure 12 exhibits that the test voltage drops from 16 V in a 5% gradient to 15.2 V. It stays for 5 s and rises to 16 V, holding at least 10 s. Functional testing is also conducted. The process is repeated until the voltage drops to 0 V. Then, the voltage is raised to 16 V. At this point, the functional level status meets C level. During the test, the BTMS controller does not work. After the test, the BTMS controller is functional. According to Article 4.6.2 of GB/T28046.2–2011, the reset function test results of the controller’s voltage dip are deemed satisfactory.

## Discussion

The experimental results demonstrate the heat dissipation capability of CSGP in BTM. It is observed that the temperature change of the battery module without CSGP at different discharge rates is different, and the high discharge rate causes the battery temperature to rise rapidly, even beyond the safe range. This emphasizes the importance of heat dissipation in the case of high-rate discharge to avoid compromising battery life and safety. Nevertheless, after the introduction of CSGP, the temperature of the battery module drops significantly under natural convection conditions, especially at the 2C discharge rate. The maximum temperature can be controlled within the safe range. In addition, the cooling effect at 3C and 4C discharge rates is also good, and the temperature curve changes smoothly. This reveals the excellent heat transfer performance of CSGP as a thermal conductivity material, which effectively improves the heat dissipation problem of the battery. Although in some cases the application of CSGP may cause temperatures to exceed the optimal range, overall, the experimental results indicate the potential of CSGP to solve the problem of battery overheating at high-rate discharge. Future research could further explore ways to optimize the use of CSGP to achieve optimal heat dissipation in various operating conditions and ensure a balance between battery performance and safety. These results provide a valuable reference for the improvement of BTMS, and also offer useful guidance for the development of new energy vehicle battery technology in the future.

## Conclusion

In previous research on BTM, the material selection of automotive BTMS has also been progressing toward energy saving. Hence, it is necessary to promote the development of new energy vehicles and introduce new materials into BTMS. However, previous related research focuses mainly on the material selection of automotive power batteries. There is little research on the thermal management performance of batteries. Thereby, new materials are introduced into the BTMS to promote more environmentally friendly and energy-saving vehicles to promote the development of new energy vehicles. On the one hand, this study proposes the working principle and thermal management scheme of a new energy vehicle battery. On the other hand, the thermal management performance of automotive batteries based on CSGP is tested. In addition, innovative designs are made in the hardware and software aspects of the BTMS controller to realize the thermal management performance of automotive batteries. This design can effectively improve the thermal management performance of automotive batteries. Results depict that: (1) After the introduction of CSGP into the automotive power battery, the temperature characteristics of the battery changed significantly, and its heat dissipation capacity became stronger; (2) A voltage sag reset function test is performed on the BTMS, the BTMS controller functions normally, and the test result is judged as qualified. The disadvantage is that the set experimental environment and parameters are carried out under ideal conditions. Other special circumstances are not considered. Consequently, the experimental results will be further improved. Different experimental scenarios will be built to evaluate BTMS further. This study is significant for further research on new materials for new energy vehicle thermal management systems.

## Data Availability

All data generated or analysed during this study are included in this published article [and its supplementary information files].If someone wants to request the data from this study please contact the Corresponding author. (Guodong Wang*, wanggd0221@163.com).
